# Diagnoses from lung specimen collected through flexible bronchoscopy from patients in a tertiary hospital in Dar es Salaam Tanzania: a retrospective cross-sectional study

**DOI:** 10.1186/s12890-019-0972-x

**Published:** 2019-11-14

**Authors:** Deus A. Ndilanha, Grace A. Shayo, Ramadhan Hassan, Moses Byomuganyizi, Leonard E. K. Lema

**Affiliations:** 1 Department of Internal Medicine, Muhimbili University of Health and Allied Sciences, P.O Box 65001, Dar es Salaam, Tanzania; 2 Department of Surgery, Muhimbili University of Health and Allied Sciences, P.O Box 65001, Dar es Salaam, Tanzania

**Keywords:** Flexible bronchoscopy, Bronchioalveolar lavage, Brush cytology, Transbronchial biopsy, Cytology, Histology, Tuberculosis, Lung tumors, Lung infections

## Abstract

**Background:**

Flexible bronchoscopy enables visualization of the respiratory airway mucosa from the oropharynx to third generation branching of the tracheobronchial tree. Bronchoscopic diagnoses vary from one locality to the other in accordance to the locality specific risk factors for lung diseases. This study aimed at describing diagnoses of all specimen of patients who underwent flexible bronchoscopy at Muhimbili National Hospital from January 2013 to November 2017.

**Methods:**

A retrospective hospital-based cross sectional study was conducted among 451 patients. Data was collected from archives and included both demographic and clinical variables. Descriptive statistics were used to summarize the study findings.

**Results:**

There was a 3 fold increase in the number of patients who underwent flexible bronchoscopy from 57 cases in 2013 to 180 cases in 2017. About 39% (174/451) of patients underwent lung biopsies while 64.5% (291/451) underwent bronchioalveolar lavage, bronchial washings or brush cytology, alone or in combination with biopsy. Generally, 64.4% (112/174) of all lung biopsies were malignant. Adenocarcinoma was the most common diagnosis seen in 33.9% (59/174). Of 76 cytological samples which were sent for bacterial culture and sensitivity, 11/76 (11.8%) were culture positive. A total of 6 (10.7%) out of 56 samples which were sent for GeneXpert MTB/RIF tested positive for *M.tuberculosis.*

**Conclusion:**

Adenocarcinoma was the most common diagnosis. Bacterial and mycobacterial infections were among the most reported findings in cytological samples. Suspicious tuberculosis lesions during bronchoscopy made it possible to diagnose tuberculosis which was hard to diagnose before patients were sent for bronchoscopy.

## Background

Flexible bronchoscopy is an endoscopic procedure that utilizes the technique of visualizing the tracheobronchial tree mucosa all the way from the oropharynx to the third generation bronchioles. It is useful both in diagnostic and therapeutic purposes [1]. The diagnostic uses include; bronchoalveolar lavage (BAL) sampling, transbronchial biopsy, bronchial washing or brushing, transbronchial needle aspiration and endobronchial ultrasound. Therapeutically flexible bronchoscopy uses include balloon dilatation, endobronchial laser ablation, electrocautery, photodynamic therapy and at times used for stent placement [1].

The advantages of flexible bronchoscopy over rigid bronchoscope is its ability to access distal airways, easily tolerated by most patients and doesn’t require general anesthesia. Flexible bronchoscope is, however not suitably applied for large and deeper biopsies [2–4].

Bronchial washing specimen obtained during bronchoscopy when coupled with other bronchoscopic sampling techniques gives complementary yield in the diagnosis of lung pathologies [5]. This is particularly so in cases like smear negative *Mycobacterium tuberculosis* and *Pneumocystis jirovecii* pneumonia (PJP) whose diagnosis in some patients has become possible only through bronchoscopic procedures [2, 6–8].

Bronchoscopic biopsy is currently the most preferred option compared to open lung biopsy due to its safety and fewer mortality rates [9–11].

Flexible bronchoscopies were done in small scale in Muhimbili National Hospital, the largest tertiary hospital in Tanzania since the year 1991. The year 2014 witnessed an intensive training mentorship of 4 young doctors which was pioneered by the Global Health Service Partnership (GHSP) in collaboration with the Muhimbili University of Health and Allied Sciences. Four other young doctors were subsequently trained making a total of 8. GHSP is dedicated to address global shortage of healthcare professionals by sending physicians and nurses to work alongside local institutes aiming at building capacities and hence strengthening the quality of medical education. Ever since, the bronchoscopy unit at the hospital became more structured and bronchoscopies are now being done more regularly than ever. Despite the presence of flexible bronchoscopic procedures for several years in the hospital, the pattern of diseases and conditions diagnosed in Muhimbili National Hospital (MNH) is largely unknown. This study aimed at describing pathological diagnoses of specimen collected from patients who underwent flexible bronchoscopy at MNH from January 2013 to November 2017.

## Methods

### Study design, site, and population

A retrospective hospital-based descriptive cross-sectional study was conducted at Muhimbili National Hospital (MNH) in Dares Salaam covering a period of 5 years from January 2013 to November 2017. Muhimbili National Hospital is the largest tertiary hospitals in the country. It is located in the Eastern Coast city of Dar es Salaam, which is also the commercial capital city of Tanzania. MNH serves as a University teaching hospital for Muhimbili University of Health and Allied Sciences (MUHAS) and other universities in the city. MNH has pulmonary units in both medical and surgical departments. The units serve patients who are admitted in the hospital and those who come on outpatient basis. Flexible bronchoscopies are done once weekly by both pulmonologists and chest surgeons. On average 3–4 patients were scoped every week. Patients who underwent bronchoscopy either came from medical pulmonology clinic, chest surgery clinic or medical and chest surgery inpatients. Some patients were referred directly for bronchoscopy from other hospitals within or outside Dar es Salaam.

The study utilized archived data of all patients who underwent bronchoscopy from January 2013 to November 2017. Bacterial culture results reported in this study had been read after 48 h of culture. Only samples of patients with suspicious lesions for tuberculosis were sent for GeneXpert MTB/RIF.

### Inclusion and exclusion criteria

Specimen which were considered for inclusion in this study had to come from adults aged 18 years or older, who underwent bronchoscopy from January 2013 to November 2017. There was no exclusion criteria.

### Data collection procedure

Records of patients who underwent flexible bronchoscopy from January 2013 to November 2017 were obtained from books in the bronchoscopy unit. This included patient’s name, hospital registration number, age, sex and indications for bronchoscopy. Hospital registration number was used to trace patient’s file for extraction of information such as residency, marital status, and confirmation of the indications for bronchoscopy. Using the Hospital registration number and patient’s name, the hospital database was accessed in search of laboratory histopathological, cytological and bacteriological results of bronchoscopic specimen. The obtained results were entered into clinical record forms (CRF).

### Data management and analysis

Data were captured into the computer using EPI Info software version 3.0. For analysis, the recorded data were transferred into SPSS version 20. Data were summarized using frequencies and percentages.

## Results

**Fig. 1:**
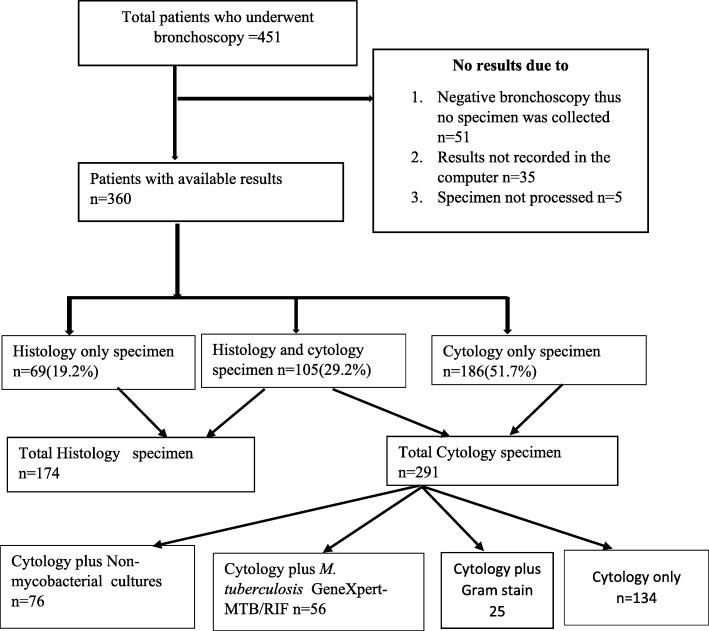
Patients’ flow and specimen collected from patients

A total of 451 patients underwent flexible bronchoscopy. Figure 1 represents patients’ flow. Majority were male, 269/451 (59.6%). The mean (SD) age of the study participants was 54.06 (15.7) years. The age range was between 18 years and 91 years. A total of 283/451 (62.7%) patients were aged 50 years or older. Majority of the patients were married 138/196 (70.4%) and were residing in Dar es Salaam 154/270(57%). Sixty eighty patients/148 (45.9%) had attained primary education as the highest ever reached education (Table 1).

**Table 1:** Socio-demographic characteristics of study patients (*N* = 451)

Characteristics	Number	Percentage
Sex		
Male	269	59.6
Female	182	40.4
Age		
18–29	34	7.5
30–39	50	11.1
40–49	84	18.6
50–59	102	22.6
60+	181	40.1
Marital Status *n* = 196^a^		
Single	30	15.3
Married	138	70.4
Widowed	18	9.2
Widower	10	5.1
Education Level *n* = 148^a^		
Primary School	68	45.9
Secondary School	38	25.7
College or above	25	16.9
No Formal Education	17	11.5
Residence *n* = 270^a^		
Dar es Salaam	154	57.0
Outside Dar	116	43.0

^a^Participants whose information on marital status, residency and/or education level were available in archives

### Trend of flexible bronchoscopy at Muhimbili National Hospital

**Fig. 2:**
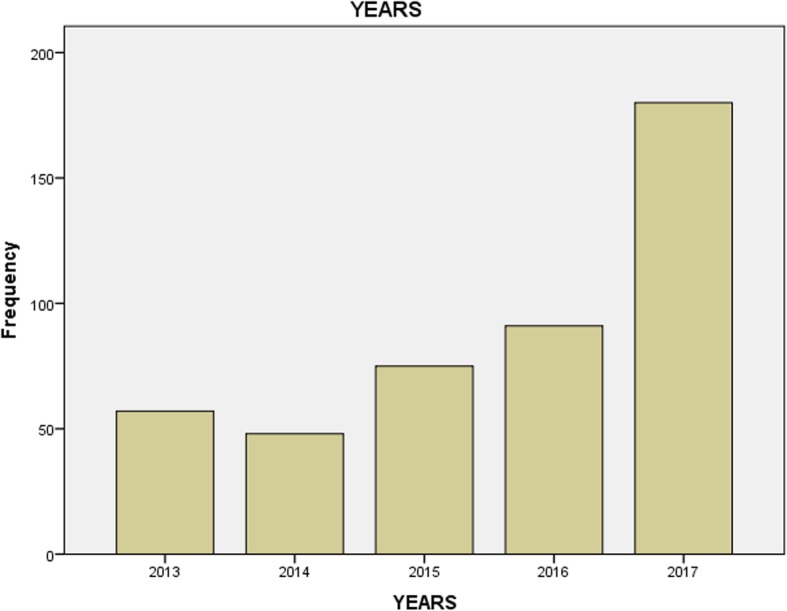
Bronchoscopy trend at Muhimbili National Hospital from 2013 to 2017

From 2013 to 2017 a total of 451 bronchoscopy were done at MNH. During this period there were gradual increase in number of bronchoscopy year after year except for the year 2014 where there was a slight drop compared to yesteryear. The year 2017 recorded the highest number of patients who underwent bronchoscopic procedures. Comparatively there were 57 patients who underwent bronchoscopy in 2013 whereas there were 180 patients who underwent bronchoscopy from January to November 2017, a more than 3 fold increase from the 2013 annual number. (Fig. 2).

### Indications for bronchoscopy for all patients who underwent bronchoscopy at Muhimbili National Hospital from 2013 to 2017

Bronchoscopy indications were lung tumors in 305/451(67.6%) patients, bronchiectasis 24 /451(5.3%), unexplained hemoptysis 21/451(4.7%), mediastinal mass 16/451(3.5%), cavitary lung diseases 16/451(3.5%), malignant-effusion15/451(3.3%), aspergillosis15/451(3.3%),

Pneumoconiosis14/451(3.1%), unexplained lung collapse 10/451(2.2%), empyema locules 7/451(1.6%), atypical pneumonia 5/451(1.1%) and exploratory bronchoscopy in 3 patients (0.7%) (Table 2).

**Table 2:** Indications for Bronchoscopy for all patients who underwent flexible bronchoscopy at Muhimbili National Hospital from 2013 to 2017

Indication	Number	Percentage
Lung tumour	305	67.6
Bronchiectasis	24	5.3
Hemoptysis-unexplained	21	4.7
Mediastinal Mass	16	3.5
Cavitary lung diseases	16	3.5
Malignant Effusion	15	3.3
Aspergillosis	15	3.3
Pneumoconiosis	14	3.1
Lung collapse	10	2.2
Empyema locules	7	1.6
Atypical pneumonia	5	1.1
Exploratory bronchoscopy	3	.7
Total	451	100.0

Table 3 summarizes histological findings of 174/451 patients who underwent tissue biopsy. Of the 174 biopsies done, adenocarcinoma was the most common diagnosis seen in 59/174(33.9%). Nonspecific acute/chronic inflammation was seen in 37/174(21.3%), squamous cell carcinoma 26/174(14.9), carcinoma not otherwise specified 16/174(9.2%) and small cell carcinoma 10/174 (5.7%). A total of 13/174(7.5%) samples were deemed inadequate and thus did not yield any results. Sarcoidosis, Aspergillosis and *M. tuberculosis* were the least found histological findings accounting to 1.7, 1.1 and 1.1% respectively. One case of Kaposi’s sarcoma was reported (0.6%) (Table 3).

**Table 3:** Histological findings for patients who underwent flexible bronchoscopic lung biopsy at Muhimbili National Hospital from 2013 to 2017, *n* = 174

Histopathological diagnosis	Number	Percentage
Adenocarcinoma	59	33.9
Nonspecific Acute/Chronic Inflammation	37	21.3
Squamous cell carcinoma	26	14.9
Carcinoma not otherwise classified	16	9.2
Inadequate sample	13	7.5
Small cell lung carcinoma	10	5.7
Normal respiratory tissue	5	3.0
Sarcoidosis	3	1.7
Aspergilloma	2	1.1
Tuberculosis	2	1.7
Kaposi’s sarcoma	1	0.6
Total	174	100.0

### Cytological findings for all patients with Bronchoalveolar lavage, brush cytology or bronchial washing from 2013 to 2017

A total of 291 patients out of 451 underwent fluid sampling for cytological analysis. The respiratory fluid samples were either collected through bronchoalveolar lavage or bronchial washing or brush cytology. Majority (199/291, 68.4%) of the findings were nonspecific acute/chronic inflammation. There were 45/291(15.5%) cytological samples which were labeled acellular. Malignancy was found in 28/291(9.6%) of fluid specimen, normal respiratory cells were reported in 11/291(3.8%). *M. tuberculosis* 3 (1.0%) and fungal infection 5(1.7%) were the least findings of all fluid samples reported (Table 4).

**Table 4:** Cytological findings for all patients with Bronchoalveolar lavage, brush cytology or bronchial washing from 2013 to 2017 (*n* = 291)

Cytological finding	Number	Percentage
Non Specific Chronic Inflammation	199	68.4
Acellular smear	45	15.5
Malignant Smear	28	9.6
Normal respiratory cells	11	3.8
Fungal infection^a^	5	1.7
Tuberculosis	3	1.0
Total	291	100.0

^a^Candida species, Aspergillus species

### Bacteriological findings among patients who underwent bronchoalveolar lavage, brush cytology or bronchial washing (n = 157)

A total of 157 samples were taken for bacteriological analysis out of 291 fluid specimens. A total of 25/157(15.9%) underwent gram staining. There was a predominance of gram positive cocci in 15/25(60%) patients while gram negative cocci and bacilli were seen in 16% each. Among the cytological samples sent for bacteriological analysis, 76 samples were sent for culture analysis, no growth was reported in 49/76(64.5%), normal flora was reported in 18/76 (23.7%). *Pseudomonas aeruginosa* was reported in 4/76 (5.3%), *Streptococcus pneumoniae* was reported in 3/76 (3.9%) while *Escherichia coli* was found in 2(2.6%).

A total of 56 samples were sent for *Mycobacterium tuberculosis* diagnosis through Gene Xpert MTB/RIF molecular technique. Of these, 6(10.7%) samples detected *Mycobacterium tuberculosis* (Table 5).

**Table 5:** Bacteriological findings among patients who underwent bronchoalveolar lavage, brush cytology and/or bronchial washing (*n* = 157)

Laboratory findings	Frequency (n)	Percent (%)
Gram stain(*n* = 25)		
Gram positive		
Bacilli	2	8
Cocci	15	60
Gram negative		
Bacilli	4	16
Cocci	4	16
Non MTB Bacterial Culture (*n* = 76)		
No Growth	49	64.5
Normal Flora	18	23.7
*S. pneumoniae*	3	3.9
*P. aeruginosa*	4	5.3
*E.coli*	2	2.6
GeneXpert MTB/RIF (*n* = 56)		
*M. tuberculosis not detected*	50	89.3
*M. tuberculosis* detected	6	10.7

*MTB Mycobacterium tuberculosis*, *RIF* Rifampicin

## Discussion

This study has demonstrated a trend of progressive increase in the number of patients who underwent bronchoscopic procedures at Muhimbili National Hospital (MNH). This trend is attributable to availability of trained bronchoscopists. It was found that majority of the patients who underwent flexible bronchoscopy in the successive period of 5 years were male aged 50 years or older, signifying that old men are the predominant candidates for flexible bronchoscopic procedures in MNH.

It was unfortunate that results for 35 patients were not traceable while 5 specimen were not processed at all after having been labeled inadequate for analysis. With increasing number of bronchoscopic procedures, there should be increased keenness in handling patients’ specimen and results.

Lung tumors were the leading indications for flexible bronchoscopy in MNH, similar to studies in India [12] and Egypt [13]. Generally, about 2/3 (63%) of patients had a diagnosis of lung malignancy. This finding concurs with findings from other studies that found lung malignancies in 64–82% of patients who underwent bronchoscopy [14]. Lung biopsies revealed that adenocarcinoma was the most common lung cancer followed by squamous cell carcinoma, carcinoma not otherwise classified, and small cell carcinoma. The predominance of adenocarcinoma in this study is similar to other recent study findings carried in United States of America, Norway and Japan where the most common malignancy was found to be adenocarcinoma, followed by squamous cell carcinoma and undifferentiated malignancy [14–16]. The study findings also tallies with recent European countries cohort studies which found an increase in the proportion of patients with adenocarcinoma among all histological subtypes [17–19]. There are a number of theories that have been put forward to explain the changes from Squamous cell carcinoma as the predominant lung malignancy up until 1980’s and the rise of adenocarcinoma as the predominant lung malignancy in the recent years in studies held in developed countries. One hypothesis is the use of low tar cigarette filters and taking large puffs to attain a satisfactory desire. These two consequently cause escapement of small particles deeper into the periphery of the lungs and thus the formation of adenocarcinoma [18, 19]. Furthermore filtered cigarettes are said to contain large amounts of nitrates. Nitrates have been found in large amounts in induced adenocarcinomas in animal studies. Air pollution especially of oxides of nitrogen has been attributed to 3% of lung cancers [2, 16, 19]. Unexplained hemoptysis and pneumonia were among the most common indications for bronchoscopy in the present study, both have also been reported as indication for flexible bronchoscopy in other studies [2, 20, 21].

Majority (68.4%) of the specimen of bronchoalveolar lavage and bronchial washings revealed inflammatory process, a finding similar to a study in Greece [21]. This finding suggests that probably most of these patients could benefit from the use of antibiotics, however this supposition doesn’t refute the fact that malignancy process almost always predisposes a person to infection.

Cytological samples identified malignancy in about 10% of patients. This low percentage might be due to the fact that very distal lesions might be hard to reach as well as fewer cytology samples were from brush cytology.

Fungi and *M. tuberculosis* infections were also picked in low percentages by the cytological samples. The low percentage for *M. tuberculosis* could partly be due to the fact that at MNH patients sent for bronchoscopy needed to be free of active tuberculosis, thus patients who underwent bronchoscopy were actively screened for active tuberculosis and declared tuberculosis free before the procedure was done. Furthermore, not all cytological samples were sent for *M. tuberculosis* or microbiological tests unless a clinician doing bronchoscopy had a suspicion for active TB infection. One study that aimed to diagnose tuberculosis through bronchoscoalveolar lavage (BAL) and transbronchial lung biopsies among patients with high risk for HIV infection isolated tuberculosis in 48% of the cases [22]. A study in South Africa by Khan et al revealed that multiple TB microbiologic tests improved diagnostic yield of BAL from 47 to 74% compared to culture alone [23]. We believe that if BAL was subjected to other microbiologic tests in conjuction with GeneXpert more TB could have been diagnosed. Thus, expanding bronchoscopy services in Tanzania will help in the diagnosis of complex lung diseases including TB diagnosis among immunodeficient patients.

## Conclusion

This study has demonstrated that bronchoscopic procedures have been increasingly performed at Muhimbili National Hospital in the past 5 years. Lung tumor was the leading indication for bronchoscopy. Adenocarcinoma was the most diagnosed malignancy. Bacterial and mycobacterial infections were among the most reported findings in cytological samples. This finding suggests a consideration of bronchoscopy for TB diagnosis in suspected patients in whom other diagnostic tests have turned negative. Screening for *M. tuberculosis* should be aggressive including sputum induction in those who can’t produce sputum prior to bronchoscopy to minimize number of TB patients in the bronchoscopy room.

### Study limitation

This was a retrospective study which solely depended on archived data thus inherent limitations like missing data or specimen were common. This might in a way have affected the results.

## Data Availability

The datasets generated and/or analyzed during the current study are not publicly available due to its liability of breaking patients’ confidentiality but are available from the corresponding author on reasonable request.

## Abbreviations

BAL: Broncho alveolar lavage
HIV: Human immunodeficiency virus
MNH: Muhimbili National Hospital
MTB: *Mycobacterium tuberculosis*
MUHAS: Muhimbili University of Health and Allied sciences
RIF: Rifampicin
TB: Tuberculosis
